# Faster ≠ Smarter: Children with Higher Levels of Ability Take Longer to Give Incorrect Answers, Especially When the Task Matches Their Ability

**DOI:** 10.3390/jintelligence11040063

**Published:** 2023-03-29

**Authors:** Martin Tancoš, Edita Chvojka, Michal Jabůrek, Šárka Portešová

**Affiliations:** 1 Psychology Research Institute, Faculty of Social Studies, Masaryk University, 602 00 Brno, Czech Republic; 2 Departments of Methodology and Statistics and Interdisciplinary Social Science, Faculty of Social and Behavioral Sciences, University of Utrecht, 3584 CH Utrecht, The Netherlands

**Keywords:** response time, distance–difficulty hypothesis, Thissen’s model, F > C phenomenon, game-based assessment, fluid intelligence, balance beam task, IRT

## Abstract

The stereotype that children who are more able solve tasks quicker than their less capable peers exists both in and outside education. The F > C phenomenon and the distance–difficulty hypothesis offer alternative explanations of the time needed to complete a task; the former by the response correctness and the latter by the relative difference between the difficulty of the task and the ability of the examinee. To test these alternative explanations, we extracted IRT-based ability estimates and task difficulties from a sample of 514 children, 53% girls, M(age) = 10.3 years; who answered 29 Piagetian balance beam tasks. We used the answer correctness and task difficulty as predictors in multilevel regression models when controlling for children’s ability levels. Our results challenge the ‘faster equals smarter’ stereotype. We show that ability levels predict the time needed to solve a task when the task is solved incorrectly, though only with moderately and highly difficult items. Moreover, children with higher ability levels take longer to answer items incorrectly, and tasks equal to children’s ability levels take more time than very easy or difficult tasks. We conclude that the relationship between ability, task difficulty, and answer correctness is complex, and warn education professionals against basing their professional judgment on students’ quickness.

## 1. Introduction

We hear school men very authoritatively saying that the fast students make the best grades and the slow ones the poorest. Statements of this kind are usually based on the assumption that if a student knows the subject in which he is being tested it should follow that he requires but a short time to make his answer. Needless to say, this assumption merits confirmation

(Longstaff and Porter 1928, p. 638; as cited in Gernsbacher et al. 2020).

It is highly likely that, when asked to imagine a straight-A student, many teachers would picture a kid who can answer all their questions without delay and always raises their hand first. This stereotype is so firm that it has paved the way into the common language. Many synonyms for the word ‘clever’ have something to do with speed: ‘quick’, ‘nimble’, or ‘fly’ are but some examples of this.

If the stereotype was valid, it could turn into a good heuristic for identifying gifted pupils or students that may be challenged to fulfil the study requirements. However, adhering to the stereotype may be dangerous when shown to be invalid, as teachers’ beliefs influence classroom practice (Brighton 2003; Cross 2009; Savasci and Berlin 2012). A significant amount of literature tackles the stereotype in mathematics. For example, Seeley (2009, 2016) claims that many teachers have internalised the stereotype. As a result, they see fast recall and computation as signs of high mathematical achievement and often neglect conceptual understanding. Stipek et al. (2001) found that maths teachers with more traditional values (mathematics being a solid body of knowledge that can be efficiently mastered without knowing what the symbols the pupils deal with represent; Thompson 1992) emphasised speed in completing a task as a necessary condition for being good at maths. When Thompson’s study was published, most American maths teachers held such traditional values. Independently of the ability in question, gifted children should be more likely to achieve above-average results. Many definitions of giftedness also include a time component (see, e.g., Subhi-Yamin 2009). Similarly, scales for giftedness ratings often have items that ask how quick and efficient the child is when dealing with a task (Pfeiffer and Jarosewich 2003; Renzulli 2021; Ryser and McConnell 2004).

Similar to Longstaff and Porter (1928; as cited in Gernsbacher et al. 2020), we think that the assumption merits confirmation even today. Most of the research carried out in the area is quickly becoming outdated. Moreover, some approaches to teaching and learning that are at odds with the stereotype (e.g., constructivist learning) are becoming more prevalent in education (Gravemeijer 2020; Steffe and Thompson 2000; Voskoglou and Salem 2020). To inspect the issue further, we may approach it from a psychological point of view.

### 1.1. The Uncertain Role of Intelligence

One of the most prominent frameworks for explaining the relationship between abilities and the time needed to complete a particular task is the Cattell–Horn–Carroll (CHC; Schneider and McGrew 2018) theory of intelligence. The CHC partitions general intelligence into three layers: the general intelligence itself (*g*; as the third-order factor), broad intellectual abilities (as second-order factors), and, finally, narrow intellectual abilities (as first-order factors). This common factor model (CFM; van Bork et al. 2017) implies that the correlation between processing speed and quantitative reasoning (ranging from 0.21 to 0.42, in the current technical manual of the WJ IV Test of Cognitive Abilities depending on age and specific subtests; McGrew et al. 2014) has arisen due to *g*. Thus, children that are both fast and capable simultaneously are so because of their general intelligence. Alternatively, Jensen (2011) equates *g* to the periodicity of neural oscillation. As such, differences in Jensen’s *g* would manifest through differences in reaction times. Therefore, both Jensen’s theory and CHC predict that children with higher levels of ability will take less time to finish a task, albeit with different mechanisms.

Nevertheless, alternative frameworks exist for modelling and understanding intelligence and related abilities. van der Maas et al. (2006, 2021) have shown that intelligence may be a dynamic system that emerges through mutual causal interactions of its components. Dynamical systems have been modelled as networks of partial correlations (or their equivalents). This allows for uncovering direct and indirect dependencies between the individual variables. This way, processing speed and particular abilities can still correlate, yet this dependency could only be induced by relationships with other variables in the system. Kan et al. (2019) compared factor and network models computed on subtests from WAIS-IV (Wechsler 2008). They found not only that the mutualistic network model explained the data better, but also that scores from the arithmetic reasoning subtest were weakly related only to one of the processing speed subtests (symbol search) and even had a direct negative relationship (albeit very small) with another processing speed subtest (coding). The mutualistic model tells a different story—students who are both fast and capable can be so for two reasons. Firstly, there is a direct relationship between processing speed (or some of its facets) and particular ability. Secondly, the students are both fast and capable because they are also good at another (so-called bridging) ability. Such a bridging ability would connect processing speed with the ability in question.

Regardless of the overarching theoretical framework, the character of the task might moderate the relationship between people’s ability levels and their response times. Goldhammer et al. (2014) showed that, when solving complex problems, students who take longer also perform better. However, when dealing with routine tasks (like basic reading), students who take longer are likely to be less successful than the faster ones. Scherer et al. (2015) observed that students who spend more time also tend to score higher on complex problem solving. To sum up, there is evidence against a direct relationship between the ability to process cognitive challenges quickly and other abilities relevant to the educational context.

### 1.2. Refuting the Stereotype

The relationship of cognitive speed with either intelligence or other more specific abilities is not straightforward and may be relatively challenging to uncover. Firstly, it is difficult to define what ‘an ability’ really is. Many theories aim to explain the same phenomenon but define abilities in vastly different ways (see the difference above between the CHC and Jensen’s *g*, where the former is an ability, yet the latter one ‘only’ causes variance in abilities). Consequently, it is not easy to obtain a good proxy for abilities. Grades do not explain enough variance in abilities on their own (Cucina et al. 2016), and standardised ability tests are not a common part of the curriculum. One way of empirically obtaining ability and difficulty estimates is via the item response theory (IRT; de Ayala et al. 2022). IRT is the most prevalent approach in modern psychometrics and is routinely used in test construction (Borsboom and Mellenbergh 2004). IRT models allow for estimating a so-called latent ability of each participant directly from answers to a set of items.

Modelling response times (through which cognitive speed is often operationalised) has a long tradition in psychology. Thurstone (1937) tried to formalise the relationship between reaction time and difficulty (defined as a ratio of people who were and were not able to perform a task successfully). van der Linden (2009) proposed a model of processing speed analogous to the model of speed in physics: processing speed equals the ratio of mental labour to time. In this paper, we will combine the two approaches. We will first use IRT modelling to extract empirical estimates of the abilities and difficulties of individual children and tasks, respectively. Then we will use these estimates to model the relationship between ability levels and the time needed to complete a task as a system of multilevel regressions.

The structure of this paper is as follows. First, we introduce the F > C phenomenon, which states that incorrect answers take more time to complete (Beckmann 2000; Beckmann et al. 1997). This phenomenon provides a conceptual basis for the model we propose. Then, we introduce Thissen’s model (Thissen 1983), which formalises the distance–difficulty hypothesis. This hypothesis states that the time needed to solve a task increases as the person’s ability nears the task’s difficulty. We then combine these two approaches in a model where the time needed to solve a task is a dependent variable. This model implies that children take the most time to solve a task whose difficulty matches their ability level, and the amount of time differs between correctly and incorrectly solved items^1^. We then test all models on data from a game-based test of logical thinking, controlling for children’s ability levels and varying task difficulties. We report how we determined our sample size, all data exclusions (if any), all manipulations, and all measures in the study (Simmons et al. 2012). We put our findings into context in the Discussion, with recommendations for future research and educational practice.

### 1.3. The F > C Phenomenon

The F > C phenomenon (Beckmann 2000; Beckmann et al. 1997) implies that incorrect responses take more time than correct ones. The F > C phenomenon can be formally expressed by Equation (1):

*t_ij_* = μ + γ *FC_ij_* + ε*_ij_*, (1)

where *t_ij_* is the response time of person *j* on item *i*, μ is an intercept (average time across each item and person), *FC_ij_* is a binary variable that indicates whether the answer to item *i* of person *j* was false or correct, γ is the unstandardised regression coefficient that could be interpreted as a mean difference in response time between the false and correct answers, and ε*_ij_* is normally distributed residuals of the model.

The F > C phenomenon is robust (Beckmann 2000; Beckmann et al. 1997; Preckel and Freund 2005; Troche and Rammsayer 2005), but, at the same time, the authors admit that the emergent difference may be an artefact of empirical aggregation, as incorrect answers are more likely when dealing with the most difficult items in a test. Moreover, Beckmann and Beckmann (2005) also suggest that the magnitude of the F > C phenomenon may differ concerning the examinee’s ability. According to the authors, people who performed worse gave incorrect responses faster than examinees who were more successful. Preckel and Freund controlled for the ability, which did not have a significant effect, though due to their relatively small sample we might also attribute this to sampling error. Our study will examine the effects of both ability and difficulty.

### 1.4. The Distance–Difficulty Hypothesis

Thissen’s model was initially proposed in 1983. It was later revised by Ferrando and Lorenzo-Seva (2007), who perceived the model as a formal representation of the distance–difficulty hypothesis. The hypothesis states that the response time for a task decreases with the distance between the person’s ability level (θ*_j_*) and the item’s difficulty (*b_i_*). In other words, people should take more time to solve tasks closer to their ability level. Conversely, people should spend less time on tasks that are substantially easy or difficult for them. Since the formal representation of the hypothesis includes an absolute value, the model implies that the predictive time differences should be the same and symmetrical. Thissen’s model is formally represented by Equation (2), which assumes a person who answers a set of items in a test within a certain time:

ln *t_ij_* = μ + τ*_j_* + β*_i_* − γ|θ*_j_* − *b_i_*| + ε*_ij_*, (2)

where ln *t_ij_* is a logarithmic transformation of the response time of person *j* spent on item *i* (the transformation is used to achieve normally distributed errors, ε*_ij_*, since the response times are assumed to be log-normally distributed); μ is the intercept, which could be interpreted as the mean time spent on all items among the whole sample; τ*_j_* is a parameter for the general speediness of person *j* (how much the person spent on the items on average); β*_i_* is the time required to answer item *i* by the person of average ability; and γ is the magnitude of the linear relationship between the ability (θ*_j_*) and difficulty (*b_i_*) absolute distance and the response time (expected to be negative by definition).

### 1.5. The Proposed Model

We will build a new model in two steps, testing two expectations. First, we will verify empirically whether the F > C phenomenon (Beckmann 2000; Beckmann et al. 1997) holds when controlling for person’s task difficulties, as the authors suggested. We expect the phenomenon to hold regardless of the item’s difficulty. We will also control for participants’ ability levels.

Secondly, the distance–difficulty hypothesis (Ferrando and Lorenzo-Seva 2007; Thissen 1983) implies that the relationship between the distance from the person’s level of ability and the time needed is symmetrical, no matter the direction of the difference. We aim to replicate this hypothesis. If both the distance–difficulty hypothesis, and the F > C phenomenon hold, we will extend Thissen’s model by the response correctness parameter (represented by the binary F > C term in Equation (1)). This parameter encodes whether the item was answered correctly. We expect this model to explain more variance than the original Thissen’s model. We will inspect whether there is a significant interaction between the ability–difficulty distance and response correctness. A significant interaction would suggest that the relationship between the time needed to solve a task and the ability-difficulty distance differs for correctly and incorrectly answered items.

## 2. Materials and Methods

### 2.1. Participants

The sample consisted of 514 children, 53% girls. The average age was 10.3 years (*SD* = 0.8 years), the youngest participant was eight years and two months old, and the oldest one was twelve years and four months old. Children were recruited in 16 Czech elementary schools willing to participate in a broader validation study of a giftedness-screening system. The schools selected the classes that would participate in the study. We worked with all children in a class whose legal guardians gave informed consent.

Since this research was a secondary data analysis, we performed an a posteriori sensitivity analysis in the *mixedpower* R package (version 0.1.0) to determine whether the sample was sufficient to detect small effects, as proposed by Kumle et al. (2021). The required sample to reach the sufficient power of 0.8 for all significant parameters in the two most complex models was 400. The setup script of our sensitivity analysis is in the Supplementary materials.

### 2.2. Measures

#### Triton and the Hungry Ocean

The data were gathered during several group sessions of pilot testing of a game-based assessment application for the identification of gifted students. The game’s concept is similar to MathGarden (Straatemeier 2014). Triton and the Hungry Ocean (referred to here as Triton) is based on the ‘balance beam task’ of Inhelder and Piaget (1958) that was later adopted by other authors and is currently referred to as Figure Weights (McGrew et al. 2014; Wechsler 2008). The objective is to choose a set of weights for one balance beam to counterbalance the weights on the other. Triton re-uses this principle in a submarine setting, including some novel features. The game uses cartoon-like graphics and simple sounds. There is no time limit for individual tasks.

**Figure 1:**
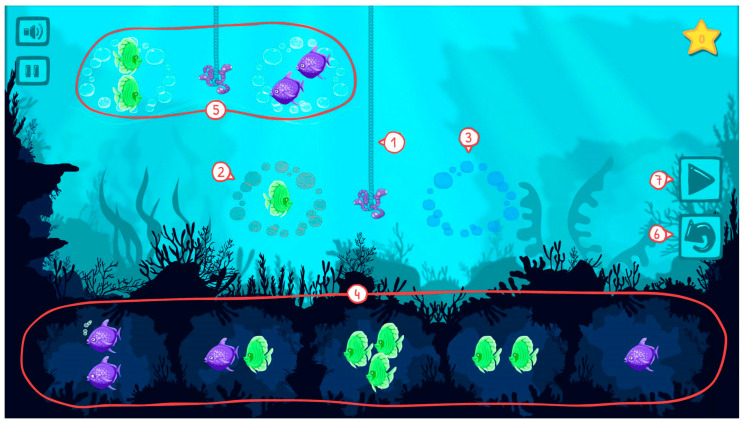
Sample task and individual game features of Triton.

A sample task is shown in Figure 1, where the individual features of the game are highlighted. Two circles are outlined by bubbles surrounding a hook (Feature 1). On the left side, the circle contains a certain number of animals (Feature 2), and the right-side circle is empty (further referred to as a slot; Feature 3). The player is supposed to fill this empty slot with one of the five groups of animals from the bottom part of the screen (Feature 4) to balance out the strength of the sea-creature group on the left side (Feature 2). Creatures of the same colour, shape, and number have the same strength. In more complex tasks, the strength of individual animals is expressed via so-called conditions: shorter hooks with both sides already occupied and balanced (Feature 5). These conditions imply the relative strength of specific animal types.

Besides moving groups of animals from the bottom part of the screen to the slots and back, the player is allowed to reset the task (i.e., return all the features to the original state) by pushing the *reset* button (Feature 6). They move to the next task by pushing the *play* button (Feature 7).

To solve the task, the player needs to deduce the relative strength of the individual animals, applying primarily logical reasoning. In general, the abilities here fall into fluid reasoning (within the CHC framework): logical reasoning intentionally and purposefully aimed at solving novel ‘on-the-spot’ problems. Such problems cannot be solved using previously learned habits, schemas, or scripts (Schneider and McGrew 2018). To solve the task, the children apply simple addition, subtraction, multiplication, and division). Therefore, they apply logical thinking within a mathematical context. This narrow ability is termed quantitative reasoning within the CHC model. However, we want to emphasise that the tasks require no advanced mathematical knowledge, and logical reasoning explains the most variance in the scores.

The game consisted of 29 tasks, the complexity of which gradually increased, as did the number of game mechanics involved. At the game’s start, three trial items did not contribute to participants’ scores. A description of these mechanics is available online in the supplemental material. A narrated video demonstration showcasing a task introduced each new game mechanic. The response time for each item was recorded from the moment children were exposed to the item task until they pushed the *play* button. In this study, we administered the game on PCs in groups of several children. Each child worked individually.

### 2.3. Data Management

We worked with data on the correctness of the solution for each task and the time spent on solving each task recorded by the *Triton and the Hungry Ocean* app. Correct responses were scored as “1”, and false ones as “0”. Response time was recorded in seconds with 0.5 s intervals. We also collected the participants’ data on gender, age, grade, and the school they attended.

No participants were excluded due to missing answers. All participants answered 22 tasks; even the last task, 29, had only 3.5% (*n =* 18) of missing values. All tasks left unanswered were coded as incorrect, and the corresponding time records were left missing.

### 2.4. Analysis plan

#### 2.4.1. Preliminary IRT Models

We first needed to establish a well-fitting IRT model using the item response correctness data as an input. This IRT model allowed us to extract children’s ability levels (θ*_j_*) and each item’s difficulty parameter (*b_i_*). We initially estimated the dichotomous Rasch model (Bond and Fox 2013) in R version 4.2.2 (R Core Team 2021) using package *mirt* (version 1.37.1; Chalmers 2012). This model estimates the probability of solving an item as a function of the participant’s ability. The dichotomous Rasch model is defined by Equation (3) as:

(3) $$P_{ij}=\frac{e^{\;\left(\theta_{j\;}{-\;b}_{i}\right)}}{{1+e}^{\;\left(\theta_{j}-{\;b}_{i}\right)}},$$

where *P_ij_* is the probability of the correct answer of person *j* to item *i*, θ*_j_* is the ability of person *j*, and *b_i_* is the difficulty parameter of item *i*.

However, the Rasch model (Bond and Fox 2013) did not fit the data well. Since the design of Triton is based on selecting at least one answer from a set of five options, it is possible to select a correct answer by guessing. We extended the model by fixing the guessing parameter (the lower asymptote of the item characteristic curve) of each task by a reciprocal of all its possible solutions (e.g., if the task has five possible solutions, the guessing parameter would be fixed to value 0.2). Fixing the parameter led to the so-called dichotomous quasi-Rasch model with guessing (Linacre 2002), defined by Equation (4) as:

(4) $$P_{\mathrm{ij}}=c_{i}+(1-c_{i})\;\frac{e^{\;\left(\theta_{j}{-b}_{i}\right)}}{{1+e}^{\;\left(\theta_{j}{-b}_{i}\right)}},$$

where *P_ij_* is the probability of the correct answer of person *j* to item *i*, θ*_j_* is the ability of person *j*, *b_i_* is the difficulty parameter of item *i,* and *c_i_* is the guessing parameter of item *i.*

This model had an acceptable fit. Thus, we extracted every item’s difficulty parameter (*b_i_*) and the ability parameter (θ*_j_*) of every participant under this model and used these in the main analyses.

#### 2.4.2. Main Analyses

We built two series of nested linear multilevel regression models. The first set of models tested the validity of the F > C phenomenon and controlled for task difficulty and participants’ ability levels. The second set tested the distance–difficulty hypothesis and its interaction with the F > C phenomenon. We used the *lme4* (version 1.1-31; Bates et al. 2015) R package to estimate these models.

First, we defined a null model (Model 0) as a baseline for both series. Model 0 included only fixed and random intercept terms for participants and items to reflect that all observations were nested in participants who answered the same items. All models in the series used logarithmic transformation of response time as a dependent variable. This transformation linearised the relationship between the predictors and the response time. This is one of the main changes from previous studies on the F > C phenomenon. Beckmann et al. (Beckmann 2000; Beckmann et al. 1997; Beckmann and Beckmann 2005) did not check distributional assumptions and used the absolute item response time. This is problematic, as their analyses assumed normal distribution, but response times are log-normally distributed (van der Linden 2009). They also did not include any random parameters in their studies, which would allow for modelling systematic differences between the items and participants answering the same set of items.

The first model series focused on the F > C phenomenon. At first (Model A1), we included only a binary predictor for answer correctness (*FC_ij_*). In the next step (Model A2), we added the predictors of item difficulty (*b_i_*) and person’s ability (θ*_j_*) as control variables. Finally (Model A3), we added the interaction term of the answer correctness and the person’s ability to investigate whether the effect of the F > C phenomenon increases or decreases with higher/lower levels of participant’s ability. Model A3 is represented by Equation (5):

ln *t_ij_* = μ + τ*_j_* + β*_i_* + γ_1_ *FC_ij_* + γ_2_
*b_i_* + γ_3_ θ*_j_* + γ_13_ *FC_ij_* θ*_j_* + ε*_ij_*, (5)

where ln *t_ij_* is a logarithmic transformation of the response time of person *j* on item *I*; μ is the fixed intercept (response time of average-ability person spent on average-difficulty item); τ*_j_* is the random intercept for each person (general speediness of each person); β*_i_* is the random intercept for each item (average time required to answer each item)*;* γ_1_, γ_2_, γ_3_, and γ_13_ are the fixed effects of corresponding predictors; and ε*_ij_* is normally distributed residuals. The previous models, A1 and A2, could be derived from this equation by setting select regression coefficients to zero (see Appendix A).

Based on one reviewer’s suggestion, we estimated an additional Model A4, which included the interaction of a person’s ability and an item difficulty besides all terms from Model A3, as an exploratory feature. This interaction term tests whether the relationship between the children’s ability and response time varies according to the item’s difficulty. The equation of this model is in Appendix A.

The second model series assessed the distance–difficulty hypothesis and its incremental validity over the F > C phenomenon. To test the distance–difficulty hypothesis, we included only the absolute difference between the child’s ability and the difficulty of each item (|θ*_j_* − *b_i_*|) in the first model (Model B1). We extended the second model (Model B2) by a binary variable representing the F > C phenomenon (*FC_ij_*) to assess the incremental validity of both concepts against each other. The model series ended with the last model (Model B3), where we added the interaction term of the distance and answer correctness (*FC_ij_*). By including this term, we examined whether the distance difficulty effect followed a different pattern with correct and incorrect responses. Model B3 is represented by Equation (6) as follows:

ln *t_ij_* = μ + τ*_j_* + β*_i_* + γ_4_ |θ*_j_* − *b_i_*| + γ_1_ *FC_ij_* + γ_14_ *FC_ij_*|θ*_j_* − *b_i_*| + ε*_ij_*, (6)

where ln *t_ij_* is a logarithmic transformation of the response time of person *j* on the item *i*; μ is the fixed intercept; τ*_j_* is the random intercept for each person; β*_i_* is the random intercept for each item*;* γ_1_, γ_4_, and γ_14_ are the fixed effects of corresponding predictors; and ε*_ij_* is a normally distributed residual^2^. The previous models, B1 and B2, could be derived from this equation by setting select regression coefficients to zero (see Appendix A).

## 3. Results

### 3.1. Ability Estimates

As previously mentioned, we first estimated the dichotomous Rasch model (Bond and Fox 2013) to obtain children’s ability (θ*_j_*) and item difficulty (*b_i_*) parameters for the main analyses. The Rasch model did not fit the item data well (*M*_2_(405) = 1255.32, *p* < .001, RMSEA = 0.065, SRMSR = 0.069, TLI = 0.867, AIC = 14,200.33, and BIC = 14,327.60). The empirical reliability of the sum score was rather high (*r* = 0.847).

We circumvented the issue by modifying the model to the quasi-Rasch model with guessing (Linacre 2002). The quasi-Rasch model showed an acceptable fit (*M*_2_(405) = 888.39, *p* < .001, RMSEA = 0.049, SRMSR = 0.075, TLI = 0.924, AIC = 14,000.78, and BIC = 14,128.04). Moreover, the empirical reliability of this model, *r* = 0.871, was slightly higher than that of the previous Rasch model. Item descriptive statistics with fixed guessing parameters and estimated difficulty parameters are listed in Table A1 (in Appendix B). The parameters from this model were used in multilevel regression models.

### 3.2. Null Model

The null model, listed in Table 1 and Table 2 as Model 0, provided a baseline for all subsequent models. The fixed intercept was significant (μ = 3.16, 95% CI [3.00, 3.33]). This parameter can be interpreted as the response time of an average-ability person on an average-difficulty item. Transforming the parameter from its logarithmic form, we obtained an average response time of 23.66 s.

We also found that random intercept terms (participants and items) explained a significant proportion of response time variance. The differences between the item intercept (var(β*_i_*) = 0.20, 95% CI [0.12, 0.33]) explained more variance of the response time than the individual differences of children in that characteristic (var(τ*_j_*) = 0.07, 95% CI [0.06, 0.08]).

### 3.3. Models Assessing the F > C Phenomenon

In Model A1, we included a binary predictor reflecting the correctness of the answer on an item. In accordance with the F > C phenomenon, we found that the false answers took children significantly more time than the correct answers (γ_1_ = −0.04, 95% CI [−0.06, −0.01]). However, the effect size was relatively small, and the transformed parameter indicated that the expected average difference in response times between wrongly and correctly answered items was 0.87 s.

In Model A2, the F > C phenomenon effect remained significant once controlled for item difficulty and children’s ability (γ_1_ = −0.05, 95% CI [−0.08, −0.03]). In addition, we found that response time was significantly higher in children with higher ability (γ_2_ = 0.06, 95% CI [0.05, 0.07]). On the other hand, response time did not have a significant relationship with item difficulty (γ_3_ = 0.05, 95% CI [0.00, 0.12]).

**Figure 2:**
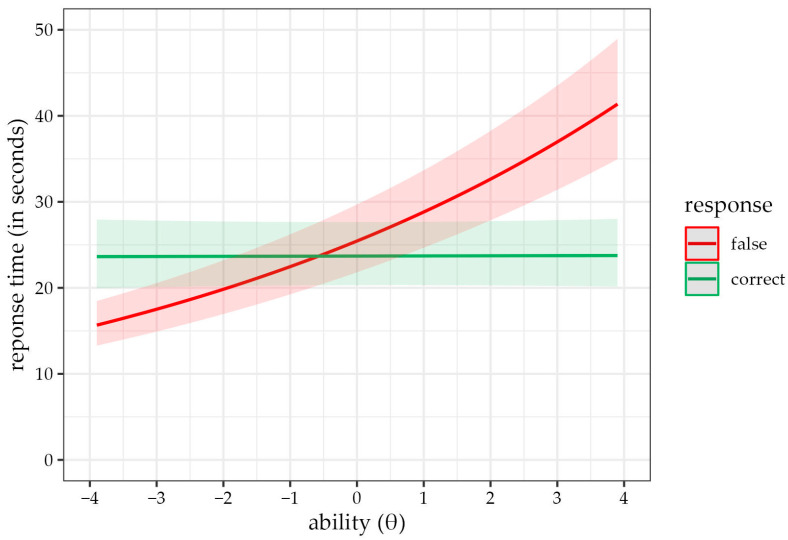
Predicted response time values according to Model A3 for correct (green) and incorrect (red) responses depending on children’s ability. With correctly answered items (green line), there is no substantial relationship between a person’s ability and the time needed to solve an item. On the other hand, with incorrectly answered items (red line), the time required to answer an item increases with the ability level. Children with greater ability, therefore, take longer to answer an item incorrectly.

In Model A3, we added an interaction of answer correctness and children’s ability, which was significant (γ_13_ = −0.12, 95% CI [−0.13, −0.11]). Adding the interaction also slightly suppressed the F > C phenomenon effect (γ_1_ = −0.07, 95% CI [−0.09, −0.04]), as well as the relationship of children’s ability with response time (γ_3_ = 0.12, 95% CI [0.11, 0.14]). All effects combined, the relationship between children’s ability and response time was negligible when the item was answered correctly. However, response time increased with higher children’s ability in case of false answers. Figure 2 illustrates these patterns.

As shown in Table 1, Model A3 was the best, and the information criteria also supported this model as the best one. All models fitted significantly better than the null model, and each model had a significantly better fit than the previous models in the sequence.

**Table 1:** Parameters of the models assessing the F > C phenomenon (interacting with a person’s ability).

		Model 0				Model A1				Model A2				Model A3			
				95% CI				95% CI				95% CI				95% CI	
	*coef.*	*est.*		LL	UL	*est.*		LL	UL	*est.*		LL	UL	*est.*		LL	UL
*Fixed effects*																	
intercept	μ	3.16	***	3.00	3.33	3.18	***	3.02	3.35	3.15	***	2.99	3.31	3.19	***	3.03	3.36
correct answer (FC)	γ_1_					−0.04	**	−0.06	−0.01	−0.05	***	−0.08	−0.03	−0.07	***	−0.09	−0.04
item difficulty	γ_2_									0.05		0.00	0.12	0.05		0.00	0.11
person ability	γ_3_									0.06	***	0.05	0.07	0.12	***	0.11	0.14
FC × ability	γ_13_													−0.12	***	−0.13	−0.11
*Random effects*																	
person intercept variance	var(τ_*j*_)	0.07	***	0.06	0.08	0.07	***	0.06	0.08	0.06	***	0.05	0.07	0.06	***	0.05	0.07
item intercept variance	var(β_*i*_)	0.20	***	0.12	0.33	0.19	***	0.11	0.32	0.17	***	0.09	0.28	0.17	***	0.10	0.29
residual variance	var(ε_*ij*_)	0.37	***	0.36	0.38	0.37	***	0.36	0.38	0.37	***	0.36	0.38	0.36	***	0.35	0.37
*Goodness of fit*																	
conditional *R*^2^					0.417				0.415				0.422				0.439
marginal *R*^2^					0.000				0.001				0.055				0.070
log-likelihood				−14,193				−14,189				−14,154				−14,008	
AIC				28,395				28,389				28,323				28,031	
BIC				28,425				28,427				28,376				28,092	
Δχ^2^ (*df*)							8.09 (1)		**		70.08 (2)		***		293.23 (1)		***

Notes. *coef.*—coefficient, *est.*—estimate, *CI*—confidence interval, *LL*—lower limit, *UL*—upper limit, *var*—variance; ** *p* < .010, *** *p* < .001.

In Model A4, we included the interaction of a person’s ability and an item difficulty. As we did not hypothesise this exploratory model, we describe and interpret it separately from Models A1–A3. As seen in Table 2, the ability–difficulty interaction was significant (γ_23_ = 0.04, 95% CI [0.03, 0.04]). This means the relationship between the ability and response time was stronger for more difficult items. Adding this information also significantly improved the model fit in comparison with the previous Model A3.

**Figure 3:**
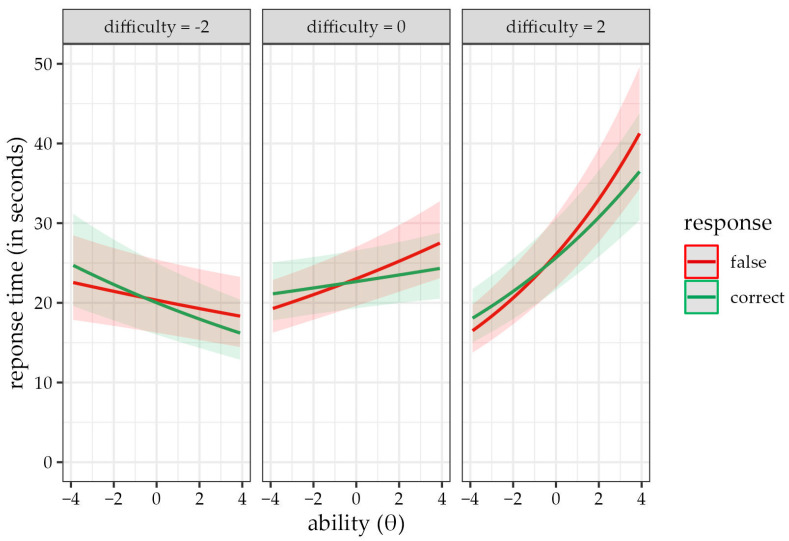
Predicted response time values according to the exploratory model A3 for correct (green line) and incorrect (red line) responses depending on children’s ability. The graph is divided into three panels based on different item difficulty levels. The observed difference in slopes of correct and incorrect responses is visibly weaker in Model A4. This further expands Model A3, as the proposition that the time required to answer incorrectly answered items (red line) increases with ability level is applicable only for moderately (e.g., difficulty = 0) and highly (e.g., difficulty = 2) difficult items.

Including the interaction term also partially explained the F > C phenomenon, whose main effect became non-significant (γ_1_ = −0.02, 95% CI [−0.04, 0.01]). The strength of the interaction between answer correctness and children’s ability also noticeably decreased (γ_13_ = −0.03, 95% CI [−0.04, −0.01]). Figure 3 aids the interpretation of the additional term. It further expands the interpretation of Model A3, indicating that the effect of the ability on time required to answer incorrectly answered items applies only to moderately and highly difficult items.

**Table 2:** Parameters of the exploratory Model A4 (including the interaction of item difficulty and person ability).

				95% CI	
	*coef.*	*est.*		LL	UL
*Fixed effects*					
intercept	μ	3.14	***	2.98	3.30
correct answer (FC)	γ_1_	−0.02		−0.04	0.01
item difficulty	γ_2_	0.06		0.00	0.12
person ability	γ_3_	0.05	***	0.03	0.06
FC × ability	γ_13_	−0.03	***	−0.04	−0.01
difficulty × ability	γ_23_	0.04	***	0.03	0.04
*Random effects*					
person intercept variance	var(τ*_j_*)	0.06	***	0.05	0.07
item intercept variance	var(β*_i_*)	0.17	***	0.10	0.28
residual variance	var(ε*_ij_*)	0.34	***	0.33	0.35
*Goodness of fit*					
conditional *R*^2^					0.466
marginal *R*^2^					0.097
log-likelihood					−13,606
AIC					27,231
BIC					27,300
Δχ^2^ (*df*)				802.28 (1)	***

Notes. *coef.*—coefficient, *est.*—estimate, *CI*—confidence interval, *LL*—lower limit, *UL*—upper limit, *var*—variance; *** *p* < .001; goodness of fit comparison with Model A3.

### 3.4. Models Assessing the Distance–Difficulty Hypothesis

In model B1, we tested the effect of absolute distance between the children’s ability and item difficulty. This effect was significant (γ_13_ = −0.13, 95% CI [−0.14, −0.12]). This means that the response time decreased 1.14 times with each logit unit of the absolute ability–difficulty distance, which is a moderately strong effect. The fixed intercept showed the average response time of 33.14 s for zero distance (the item difficulty equivalent to the person’s ability), where the time was at its maximum. The estimated response time decreases to 29.03 when the ability–difficulty distance is one logit unit, to 25.45 when the distance is two logit units, and so on.

Combining ability–difficulty distance with the F > C phenomenon led to Model B2, where the F > C phenomenon did not show a significant effect (γ_1_ = −0.02, 95% CI [−0.04, 0.01]); the ability–difficulty distance effect remained unchanged (γ_4_ = −0.13, 95% CI [−0.14, −0.12]).

**Figure 4:**
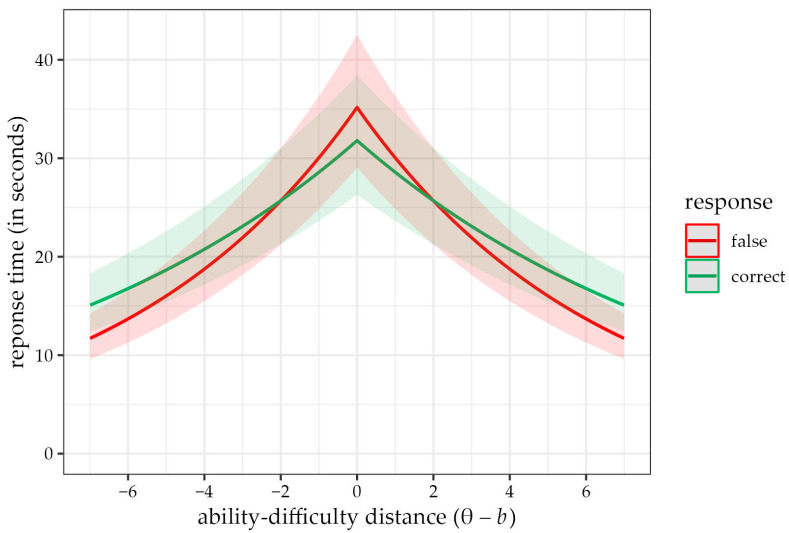
Predicted response time values according to Model B3 as a function of ability–difficulty distance separated by whether the answer was correct (green) or incorrect (red). Regardless of the response correctness, items whose difficulty matches the participant’s ability level take the longest time to solve (with incorrect answers taking the longest). The relationship changes for very difficult and easy items—with these, correctly answered items take longer than the incorrect ones.

In Model B3, we extended the previous model by the interaction of the ability–difficulty distance effect and the F > C phenomenon, which was significant (γ_14_ = 0.05, 95% CI [0.04, 0.07]). Including the interaction also suppressed the F > C phenomenon effect, which became significant (γ_1_ = −0.10, 95% CI [−0.13, −0.07]); small suppression was also visible in the ability–difficulty distance effect (γ_4_ = −0.16, 95% CI [−0.17, −0.15]). Interpretation-wise, the interaction effect means that, for false answers, the negative relationship between ability–difficulty distance and response time is stronger than for correct answers. A graphical description of these effects is in Figure 4.

This sequence of models also showed that Model B3 outperformed the preceding models in fit and information criteria—as listed in Table 3. All three models were significantly better than the null model; nevertheless, Model B2 did not fit significantly better than Model B1 (Δχ^2^(1) = 2.14, *p* = 0.144).

## 4. Discussion

In this study, we wanted to test the validity of the ‘faster equals smarter’ stereotype. More precisely, our aim was twofold. First, we wanted to inspect whether the F > C phenomenon holds when controlling for item difficulty and a person’s ability, as Beckman et al. (Beckmann 2000; Beckmann et al. 1997; Beckmann and Beckmann 2005) mentioned that the phenomenon might be a byproduct of empirical aggregation. In our data, the F > C phenomenon remained significant after controlling for ability and difficulty, albeit with a small effect size. Item difficulty did not influence the time needed to solve a task, but children’s ability did, though only with moderately and highly difficult items. Moreover, we found an interaction between ability level and correctness. Together, these results mean that children with higher levels of ability take longer to give incorrect answers on moderately difficult and difficult items than their peers with lower levels of ability. With correct answers, there is no relationship between ability and time. If the ‘faster equals smarter’ stereotype is true, it would imply that children with higher levels of ability answer more quickly in general. Our results, therefore, go against the stereotype, as they imply either no difference at all or longer response times of children with higher ability levels. 

There are several explanations for this phenomenon. High-ability children may, on average, possess better meta-cognitive skills (Swanson 1992). They may invest some time to devise a strategy before turning in an answer, regardless of its correctness. These children may also put more effort into problem solving because of their better self-regulation and thus show greater persistence (Howard and Vasseleu 2020). High-ability children are also likely to see difficult tasks as challenging. This positive framing can make them spend more time on a task, as opposed to their peers that may see difficult tasks as too demanding (Bouffard-Bouchard et al. 1993). Finally, it is worth noting that the causality can also flow in the reverse way: high-ability children may be persistent and determined in the first place, which may, in turn, accelerate their development and make them score high in ability tests. 

The small effect size may reflect the true phenomenon’s strength, as Beckman et al. (Beckmann 2000; Beckmann et al. 1997; Beckmann and Beckmann 2005) violated the distributional assumptions of their analysis, which likely inflated the estimated effect. The parametric *t*-test performed in their study assumed a normal distribution, but response times are commonly assumed to be log-normally distributed (van der Linden 2009). Moreover, in alignment with the original authors’ proposition, part of the variance in time previously explained by the correctness as the only parameter has indeed been explained by children’s ability, which attenuates the main effect of correctness.

Since the F > C phenomenon held, we proceeded to the study’s second aim: to test the distance–difficulty hypothesis and build a new model that included the relative difference between the examinee’s ability level and the task difficulty, and the F > C phenomenon. This allowed us to assess whether the phenomena were incrementally valid over one another; in other words, whether the relationship between the distance–difficulty term and the time needed to solve an item differed between correctly and incorrectly solved tasks. We supported the distance–difficulty hypothesis and found its interaction with the F > C phenomenon. This means that items that match the children’s ability take the longest to solve, and the time is even longer for incorrectly answered items. As the difference between the ability and item difficulty grows (when the examinee works with tasks that are very easy or difficult for them), the difference narrows to the point when it changes direction. Tasks that are too easy or too difficult take more time when answered correctly than incorrectly, as the hypothesis operates with the absolute difference value.

This seems intuitive with items whose difficulty surpasses the child’s ability, as solving such tasks would require considerable cognitive effort, which would ultimately increase the time needed to produce a correct answer. Since the order of the items in Triton was roughly arranged according to their difficulty, the most difficult items were administered towards the end of the test. In such a setting, children may have been frustrated, bored or tired. Experiencing the discomfort could have made them simply give up on the task, guess, or produce an erroneous solution when trying to turn the answer in as soon as possible.

However, when the tasks were far below their ability levels, children also took longer to answer correctly. As opposed to very difficult tasks, this may seem surprising. In this situation, for instance, children could have hesitated about whether the task was that easy or whether they may have overlooked something or faced a trick question, which would have made them ponder about the answer and submit it later. Moreover, the solutions to the easiest task may have seemed obvious, leading children to rush the answer and answer incorrectly. Since the easiest items were administered at the start of the test, it is also possible that some children had not understood the instructions fully and underestimated the task, leading to erroneous answers. 

It also is worth noting that the main effect of correctness only became significant once we added the interaction into the model. The interaction between the distance–difficulty term and the correctness of the answer removed irrelevant variance of the main term, strengthening its relationship with response time.

These findings may influence how we perceive time demands in educational practice. According to our results, solving tasks on newly acquired material, whose difficulty parallels a child’s ability, takes the most time. This is in line with classic theories of learning acquisition, such as Vygotsky’s proximal development zone (Eun 2019; Roth 2020), which builds upon the thesis that learning primarily happens when dealing with tasks whose difficulty is close to the child’s ability. Mediated learning experience (Tzuriel 2021) also works with the mechanism. The mediators adjust the difficulty of the task to provide the child with appropriate learning stimuli. When facing complex tasks, the mediator should offer substantial support. On the other hand, with simple tasks, the mediator should withdraw. Children that seem to take too long to solve some tasks may need this time to assimilate new knowledge. The need for time may signalise ongoing learning instead of poor performance.

To our knowledge, this is the first study that combines answer correctness and the distance between the ability and difficulty to explain variance in response times. We have shown that both the F > C phenomenon and the distance–difficulty hypothesis hold and are incrementally valid over one another. Our findings also go against the stereotype that children who are more able solve tasks quickly. However, our results must be interpreted with caution, as overall our estimated effects are not of great magnitude. Moreover, it is essential to remember that our findings concern isolated individual tasks and may differ when dealing with a whole test.

To illustrate this problem, imagine two students, Patt and Matt, and a test of 10 items ordered by difficulty. The expected response time for a correct answer (regardless of difficulty) is larger than for an incorrect answer. Patt’s ability is higher than Matt’s. Patt answers seven items before she starts struggling and giving incorrect answers that take longer. Matt already struggles with the fourth item. There are settings when Patt will finish sooner than Matt, as she takes longer to give incorrect answers but produces fewer of them. There are also configurations when the difference may be negligible. Since the effect and parameters estimated in our study were extracted directly from the test data, our results are not a universal predictive guide. The difference in the total time needed to complete a specific test between two children with different ability levels depends on the configuration of the differences between their abilities and the differences in the time needed for giving correct vs incorrect answers. We do not want to imply that children who are more able will always finish later. However, we do want to refute the expectation that a more capable child will automatically finish sooner.

Furthermore, both the F > C phenomenon and the distance–difficulty hypothesis assume that the test in question is not a timed test. Timed tests are more prevalent in the educational context, and it is reasonable to assume that, with a time limit, additional factors need to be considered, for instance, the individual strategies of the test-takers. Cultural context and school climate may play a role as well. Despite recent developments, the Czech school system has been mostly seen as authoritarian and focused on memorising (Perry 2005). It prioritises the successful transmission of knowledge over creativity and positive learning experience (Straková and Simonová 2013). Children educated in such a system may not be motivated to keep working on a task without knowing the correct answer right away. The effects we found may be less or more pronounced in different school systems; for instance, based on how the school system treats failure or how strongly the teachers have internalised the ‘faster equals smarter’ stereotype. Finally, while we think that our computational approach allowed us to remedy some methodological issues of the studies we built upon, it is not perfect. Our two-step estimation (first IRT and then multilevel regression model) leads to two sets of errors. Certain modelling approaches, such as generalised mixed modelling for explanatory item response analyses (De Boeck and Wilson 2004; Baayen et al. 2008), allow for incorporating the person- and item-level effects in a one-step estimation that also takes the clustered data structure into account. It may be interesting to re-analyse our or similar data using these techniques to get even more accurate estimates of the effects reported in our study. We hope that these limitations can inform future research on related topics.

Further research can also explore whether our results also hold with knowledge-based tasks, which would tap into crystallised, as opposed to fluid, intelligence. Teachers often observe that students who can quickly answer questions tend to have better knowledge accessibility, suggesting a strong understanding of the subject. Goldhammer et al. (2014) have shown that students who take longer perform better with complex tasks, but with routine tasks (like basic reading) the opposite is true. We should investigate and separate the contexts where we can refute the stereotype from these where it still may apply to some extent. Overall, the relationship between the time needed to solve a task and children’s ability, task difficulty, and answer correctness is complex, and the influence of other variables is yet to be examined. Nevertheless, educational professionals should avoid basing their professional judgement on how quickly a child processes a task and be aware that tasks with great learning potential are likely to take a long time.

## Figures and Tables

**Table 3:** Parameters of the models assessing distance–difficulty hypothesis interacting with the F > C phenomenon.

		Model 0				Model B1				Model B2				Model B3			
				95% CI				95% CI				95% CI				95% CI	
	*coef.*	*est.*		LL	UL	*est.*		LL	UL	*est.*		LL	UL	*est.*		LL	UL
*Fixed effects*																	
intercept	μ	3.16	***	3.00	3.33	3.50	***	3.31	3.69	3.51	***	3.32	3.70	3.56	***	3.37	3.75
correct answer (FC)	γ_1_									−0.02		−0.04	0.01	−0.10	***	−0.13	−0.07
ability–difficulty distance	γ_4_					−0.13	***	−0.14	−0.12	−0.13	***	−0.14	−0.12	−0.16	***	−0.17	−0.15
distance × FC	γ_14_													0.05	***	0.04	0.07
*Random effects*																	
person intercept variance	var(τ_*j*_)	0.07	***	0.06	0.08	0.07	***	0.06	0.08	0.07	***	0.06	0.08	0.07	***	0.06	0.08
item intercept variance	var(β_*i*_)	0.20	***	0.12	0.33	0.25	***	0.15	0.42	0.25	***	0.15	0.42	0.26	***	0.16	0.45
residual variance	var(ε_*ij*_)	0.37	***	0.36	0.38	0.34	***	0.33	0.35	0.34	***	0.33	0.35	0.34	***	0.33	0.34
*Goodness of fit*																	
conditional R^2^					0.417				0.527				0.525				0.542
marginal R^2^					0.000				0.082				0.081				0.092
log-likelihood				−14,193				−13,563				−13,562				−13,537	
AIC				28,395				27,136				27,136				27,088	
BIC				28,425				27,174				27,181				27,142	
Δχ^2^ (*df*)							1260.82 (1)		***		2.14 (1)				49.49 (1)		***

Notes. *coef.*—coefficient, *est.*—estimate, *CI*—confidence interval, *LL*—lower limit, *UL*—upper limit, *var*—variance; *** *p* < .001.

## Data Availability

The data, together with analytic scripts, are freely available on the Open Science Framework repository under the following URL: https://osf.io/dhu7f/ (accessed on 22 March 2023).

## Notes

1 In this study, we use the terms *‘item’* and *‘task’* semi-interchangeably. The word *‘item’* refers to a clearly demarcated part of the test whose psychometric difficulty can be empirically extracted. The word *‘task’* refers to the content of the item. In the case of Triton, children solve the same *‘task’* (balance both sides of the equation) many times, though are administered *‘items’* of varying difficulty.

2 Please note that the individual effects of item difficulty (*b_i_*) and a person’s ability (θ*_j_*) are not included in the model, as they are already used to form the distance–difficulty difference term.

## Supplementary Materials

The following supporting information can be downloaded at: https://www.mdpi.com/article/10.3390/jintelligence11040063/s1; Table S1: game mechanics; R script S1: Analytic script; R script S2: Plot script; R script S3: Sensitivity analysis (with results).

## Appendix A

In this section, all model equations used in this study are listed.

Null model (Model 0):

ln *t_ij_* = μ + τ*_j_* + β*_i_* + ε*_ij_*, (A1)

Model A1:

ln *t_ij_* = μ + τ*_j_* + β*_i_* + γ_1_ *FC_ij_* + ε*_ij_*, (A2)

Model A2:

ln *t_ij_* = μ + τ*_j_* + β*_i_* + γ_1_ *FC_ij_* + γ_2_
*b_i_* + γ_3_ θ*_j_* + ε*_ij_*, (A3)

Model A3:

ln *t_ij_* = μ + τ*_j_* + β*_i_* + γ_1_ *FC_ij_* + γ_2_
*b_i_* + γ_3_ θ*_j_* + γ_13_ *FC_ij_* θ*_j_* + ε*_ij_*, (A4)

Model A4:

ln *t_ij_* = μ + τ*_j_* + β*_i_* + γ_1_ *FC_ij_* + γ_2_
*b_i_* + γ_3_ θ*_j_* + γ_13_ *FC_ij_* θ*_j_* + γ_23_ *b_i_* θ*_j_* + ε*_ij_*, (A5)

Model B1:

ln *t_ij_* = μ + τ*_j_* + β*_i_* + γ_4_ |θ*_j_* − *b_i_*| + ε*_ij_*, (A6)

Model B2:

ln *t_ij_* = μ + τ*_j_* + β*_i_* + γ_4_ |θ*_j_* − *b_i_*| + γ_1_ *FC_ij_* + ε*_ij_*, (A7)

Model B3:

ln *t_ij_* = μ + τ*_j_* + β*_i_* + γ_4_ |θ*_j_* − *b_i_*| + γ_1_ *FC_ij_* + γ_14_ *FC_ij_*|θ*_j_* − *b_i_*| + ε*_ij_*, (A8)

## Appendix B

**Table A1:** Item descriptive statistics and quasi-Rasch model parameters.

				Response Correctness		Response Time (in Seconds)		
Item	Sample	Guessing	Difficulty	*M*	*SD*	*M*	*SD*	*Mdn*
item 1	514	0.200	−1.28	0.75	0.43	21.19	24.57	15.00
item 2	514	0.200	−1.91	0.81	0.39	19.52	17.80	14.50
item 3	514	0.067	−3.35	0.91	0.28	31.75	24.68	24.00
item 4	514	0.067	−3.12	0.90	0.30	20.80	21.49	16.50
item 5	514	0.050	−1.26	0.71	0.46	47.71	38.18	35.50
item 6	514	0.067	−2.32	0.83	0.37	20.07	15.59	15.50
item 7	514	0.200	−1.97	0.84	0.37	15.96	25.88	10.00
item 8	514	0.100	−2.54	0.86	0.35	24.81	21.55	18.00
item 9	514	0.200	0.17	0.59	0.49	13.52	10.57	10.00
item 10	514	0.050	−1.62	0.76	0.43	34.38	19.46	28.50
item 11	514	0.200	−0.54	0.68	0.47	22.89	18.04	17.50
item 12	514	0.200	−1.55	0.79	0.41	10.87	8.86	8.50
item 13	514	0.200	2.91	0.25	0.44	15.92	16.14	11.00
item 14	514	0.050	−0.17	0.56	0.50	51.22	30.05	44.20
item 15	514	0.200	0.99	0.49	0.50	27.23	19.43	21.00
item 16	514	0.017	0.31	0.46	0.50	50.40	33.98	42.50
item 17	514	0.200	1.40	0.44	0.50	31.63	24.05	26.00
item 18	514	0.200	1.29	0.42	0.49	25.93	20.69	20.00
item 19	514	0.050	0.35	0.48	0.50	42.44	28.52	34.25
item 20	514	0.200	1.74	0.38	0.49	42.19	32.50	33.75
item 21	514	0.200	2.19	0.32	0.47	29.14	23.76	22.50
item 22	514	0.067	3.15	0.19	0.39	61.93	53.74	45.75
item 23	514	0.200	1.44	0.40	0.49	25.45	19.60	19.50
item 24	510	0.100	3.60	0.16	0.37	43.21	36.16	31.75
item 25	507	0.050	3.09	0.15	0.36	60.81	52.38	46.00
item 26	504	0.200	4.88	0.15	0.36	39.15	42.53	26.50
item 27	502	0.200	3.71	0.28	0.45	31.27	28.12	22.50
item 28	500	0.050	5.23	0.06	0.24	44.33	37.55	33.50
item 29	496	0.017	5.80	0.04	0.19	45.70	40.60	35.00

Note. Correct response was scored as “1”, false as “0”, thus, response correctness mean represents the ratio of correct responses.
