# Supplementary material for: Faster ≠ Smarter: Children with Higher Levels of Ability Take Longer to Give Incorrect Answers, Especially When the Task Matches Their Ability
Source: J Intell. 2023 Mar 29;11(4):63. doi: 10.3390/jintelligence11040063 (PMC10143123; doi:10.3390/jintelligence11040063)
Supplement: Supplementary file 1 [file jintelligence-11-00063-s001.zip › Table S1-Game mechanics.pdf]

An overview of the individual tasks and game mechanics is shown in Table S1 below.

Table S1. Characteristics of tasks and principles of the Triton and the Hungry Ocean game.

Item	Characteristics of tasks and game principles	Visual samples of tasks
trial 1–3	Mere copying of the left side of the main hook. One must understand the main game principle—animals with the same color, shape, and number have the same strength.	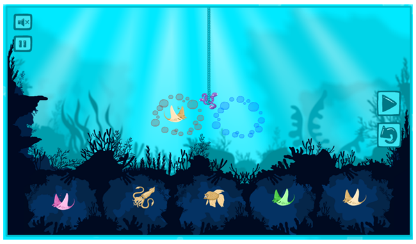
1–3	New principle of "slots". Each side of the longest hook can be divided up to four subsets (slots). One must add the strength of the separate animal groups on each side. Simultaneously, the player does not choose only one answer from the five options but fills in all the empty slots on the right side of the hook.	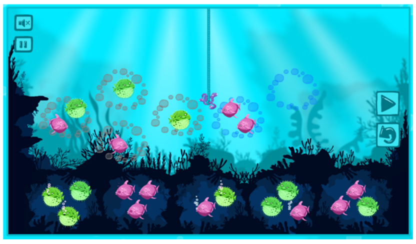
4–5	New principle: "potion with deadly water." When applied, it will eliminate all the features in one slot (sub-set) on the left side of the main hook. Animated sample is available on the link: https://bit.ly/3luKObR	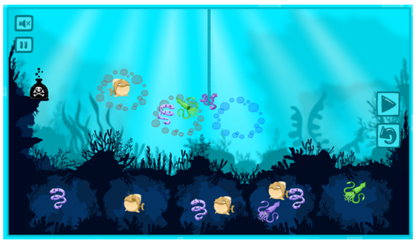
6–7	New principle: "potion with living water." When applied, the number of features in any slot on the right side of the main hook will be doubled. Animated sample is available on the link: https://bit.ly/36u7ll5	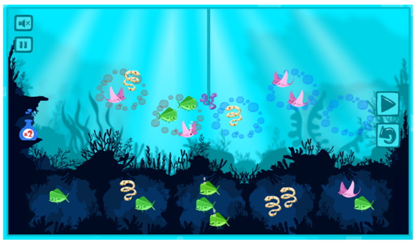

Table S1. (continued).

8–18	New principle: "1st condition." A new hook on the top left side of the screen with already balanced sides to indicate the relations between the individual animals. The task complexity gradually grows and the nature of logical operations necessary for the solution changes. In some cases, tasks involve game principles that have been already introduced (slots, both types of potions) or their combination. However, in none of the tasks are both types of potion present at once.	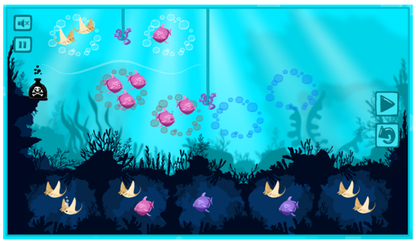
19–29	New principle: "2nd condition." A new hook on the top right of the screen with already balanced sides. One must use information from both conditions at once and apply more complex logical operations. In the tasks, game principles are used that have already been introduced before.	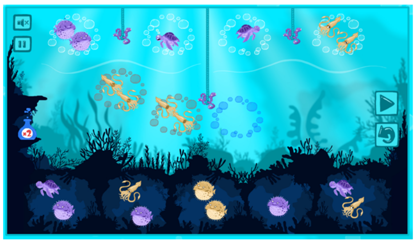
